# On the Benefit Arising from Pressure Applied to the Head, in Certain Cases of Hydrocephalus

**Published:** 1822-03

**Authors:** Thomas Girdlestone, Charles Costerton

**Affiliations:** Great Yarmouth; Yarmouth


**Art. III.—*On the Benefit arising from Pressure applied to the Head, in certain Cases of Hydrocephalus.* By T. Girdlestone, M.D. and Charles Costerton, Esq.**

THOUGH I have not the honour of being personally acquainted with Sir Gilbert Blane, I had the good fortune to profit by his ingenious paper, in the London Medical Journal of October last; as the statement which follows, from Mr. Costerton, an intelligent surgeon of this town, will evince.

THOMAS GIRDLESTONE.

*Great Yarmouth; Feb.* 1*st,* 1822.

---

Mary Monks, a married woman, was delivered of a male child, on the 3d of October, 1819.

About a fortnight afterwards, the child became ill with sickness and fever, and a constant moaning and crying, without any very apparent dilatation of the pupils of the eyes, but a considerable projection of the left parietal bone. The child visibly wasted. Small doses of calomel were administered; but, as no benefit was obtained in pursuing these means, and the parents were poor, the medicines were discontinued, and, after wasting for two months, the child died. There was no examination of the head, as I was not apprised of his death till after he was buried.

On the 9th of April, 1821, this woman was brought to-bed of another boy, apparently born in good health. About three months after, his head became enlarged about the left parietal bone, in the same manner as the child who had died.

In the beginning of last October, the mother took him to Dr. Girdlestone; and, he learning, from her, that the same appearance had been discovered in the other child before its death, and that I had delivered her of each of these children, deferred recommending any medical treatment, until he had some conversation with me. On Dr. Girdlestone seeing me, he detailed to me a paper he had just read in the London Medical Journal of October, from Sir Gilbert Blane, and gave it as his opinion that the child was a proper subject for trying the treatment recommended by that physician. This child had the same symptoms of wasting, &c. as the other who died; and, superadded to them, a rupture on each side of the body. Dr. Girdlestone, therefore, wished me to try first, without the aid of medicine, the simple effect of pressure on the brain; and, as I could not succeed in confining a bandage round the parts, I put a double strap of adhesive plaster round the head, which completely answered the purpose. The straps remained firm as they were originally fixed. The child gradually improved under the pressure; and the head, which originally was bald, began to be covered with hair, and to acquire more uniformity; and, as the muscular strength of the abdomen increased, the ruptures disappeared. The teeth are cutting; the child is growing stronger, and may be said to be nearly well.

(Signed) CHARLES COSTERTON.

*Yarmouth; January 29th,* 1822.

